# G-quadruplex structures in 16S rRNA regions correlate with thermal adaptation in prokaryotes

**DOI:** 10.1093/nar/gkaf042

**Published:** 2025-01-30

**Authors:** Bo Lyu, Kangkang Niu, Deborah Anderson, Qili Feng, Qisheng Song

**Affiliations:** Division of Plant Science and Technology, University of Missouri, Columbia, MO 65211, United States; Guangzhou Key Laboratory of Insect Development Regulation and Application Research, Institute of Insect Science and Technology, School of Life Sciences, South China Normal University, Guangzhou 510631, China; Department of Veterinary Pathobiology, University of Missouri, Columbia, MO 65211, United States; Guangzhou Key Laboratory of Insect Development Regulation and Application Research, Institute of Insect Science and Technology, School of Life Sciences, South China Normal University, Guangzhou 510631, China; Division of Plant Science and Technology, University of Missouri, Columbia, MO 65211, United States

## Abstract

G-quadruplex (G4) structure is a nucleic acid secondary structure formed by guanine-rich sequences, playing essential roles in various biological processes such as gene regulation and environmental stress adaptation. Although prokaryotes growing at high temperatures have higher GC contents, the pattern of G4 structure associated with GC content variation in thermal adaptation remains elusive. This study analyzed 681 bacterial genomes to explore the role of G4 structures in thermal adaptation. Our findings revealed a strong positive correlation between G4 patterns in the region encoding 16S rRNA genes and optimal growth temperatures (T_opt_), whereas genomic GC content and G4 patterns did not show significant correlations with T_opt_. Evolutionary analysis showed distinctive differences in G4 stability between *Thermotoga* (T_opt_ ≥ 80°C) and *Pseudothermotoga* (60°C ≤ T_opt_ < 80°C) species, with *Thermotoga* species exhibiting higher G4 stability, indicating stronger selective pressure for G4 structures. *In vitro* spectroscopy analysis showed that base mutations at key sites resulted in the absence of G4 structural stability and integrity in *Thermotoga* compared to *Pseudothermotoga*. Collectively, this study suggests that the G4 structures in 16S rRNA regions emerged as key indicators of thermal adaptation in prokaryotes and contributes to our understanding of the molecular basis of evolutionary adaptation.

## Graphical Abstract

**Graphical Abstract:**
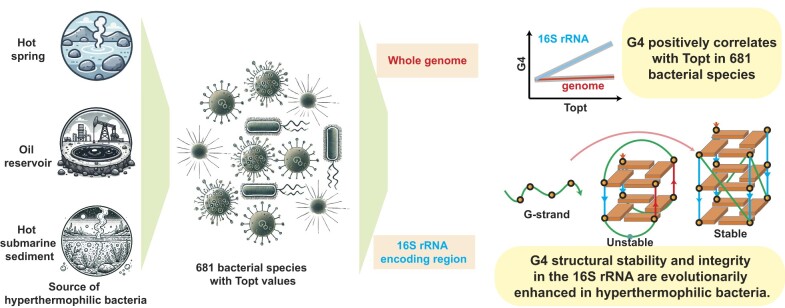

## Introduction

G-quadruplexes (G4s) are unique nucleic acid secondary structures that form in guanine-rich regions of DNA and RNA [1, 2]. These structures are characterized by the stacking of four guanine bases into a planar arrangement known as a G-tetrad, which is stabilized by Hoogsteen hydrogen bonds [3]. Multiple G-tetrads can stack on top of each other, forming a stable G4 structure [4]. The formation and stability of G4 structures are further supported by the presence of monovalent cations such as potassium or sodium, which fit closely between the G-tetrads [5, 6]. G4 structures can adopt various topologies, including parallel, antiparallel, and hybrid forms, depending on the orientation of the DNA or RNA strands and the loop arrangements connecting the G-tetrads [4, 6]. G4 structures play a crucial role in the regulation of gene expression by modulating the transcriptional activity of certain genes [7, 8]. G4 structures are also involved in the maintenance of genome stability, particularly in regions prone to genetic instability, such as telomeres and oncogene promoters [9–11]. At telomeres, G4 structures protect chromosome ends and regulate telomerase activity, thus playing a vital role in cellular aging and cancer prevention [10, 12]. Additionally, G4 structures are implicated in the replication and transcription processes, where they can act as roadblocks to polymerase enzymes or as binding sites for specific proteins that modulate these processes [13]. Current research on G4 structures has expanded significantly, revealing their widespread presence and functional importance across various organisms spanning bacteria (e.g. *Escherichia coli* and *Bacillus subtilis*), archaea, and eukaryotes, including humans [14, 15].

Thermophiles and hyperthermophiles are microorganisms that thrive in extremely high-temperature environments, with thermophiles having optimal growth temperatures (T_opt_) between 45°C and 80°C, and hyperthermophiles exceeding 80°C [16, 17]. These organisms exhibit remarkable structural adaptations that enable their survival and proliferation in such extreme conditions [18]. Their proteins and enzymes feature increased ionic bonds and hydrophobic cores that prevent denaturation at high temperatures [19, 20]. Membrane lipids of these microorganisms possess unique ether linkages, providing additional thermal stability and preventing membrane fluidity loss [21]. Additionally, thermophiles and hyperthermophiles have efficient DNA repair systems that mitigate the damaging effects of high temperatures on genetic material [18, 22]. A notable characteristic of these microorganisms is their genomic composition; it was a long debate that the genome of thermophiles and hyperthermophiles displays a high GC content, which contributes to the stability of the DNA double helix [23, 24]. Previous evidence suggests that genomic GC content correlates positively with T_opt_ within prokaryotic families, asserting that environmental factors influencing GC content evolution are less variable among closely related species [25]. This correlation was observed across 20 prokaryotic families, where the number of families showing positive correlations was significantly higher than expected by chance, irrespective of common ancestry. However, further studies updated T_opt_ values and found that positive correlations between T_opt_ and genomic GC content disappeared in some families [26, 27]. Until recently, this debate appeared to be settled by a large-scale analysis suggesting that prokaryotes thriving in high temperatures exhibit increased genomic GC contents, with thermal adaptation being a proposed reason for this positive association [28]. However, the results of this study still have limitations, as the positive correlation is weak, and it is evident that some hyperthermophiles, such as *Thermocrinis albus* (GC content 46.9%), *Thermodesulfobacterium geofontis* (GC content 30.6%), and *Thermotoga petrophila* (GC content 46.1%), do not have significantly higher GC contents compared to mesophiles [28, 29].

Few studies have investigated the relationship between G4 structures and growth temperature in prokaryotic species. One study analyzed the location of putative G4 sequences annotated genomes from the order Thermales, finding these G-rich sequences to be randomly distributed [30]. Another study speculated that there is no correlation between genomic GC content in Archaea (and Bacteria) and optimal growth temperature, likely because DNA *in vivo* is topologically closed and stable up to at least 107°C [31, 32]. Therefore, a higher density of G4-prone motifs in thermophiles due to a GC-bias is not anticipated [31]. A comprehensive study of 1627 bacterial genomes indicated that the highest frequency of G4 forming sequences was detected in the subgroup Deinococcus-Thermus, and the lowest frequency in Thermotogae [33]. These findings suggest a hypothesis that thermophilic organisms are enriched with G4s as a necessary adaptation to thermally stabilize their genomes for survival at high temperatures, with the underlying evolutionary mechanisms yet to be explored.

In this study, we analyzed 681 prokaryotic genomes from samples covering over a hundred variations of growth temperature to investigate the presence of G4 forming sequences. Our results indicate an evolutionary shift toward an increased frequency and stability of G4s in the region of genome encoding the 16S rRNA genes along the T_opt_ spectrum for the first time. This finding suggests that G4s play a significant role in the adaptation and survival of prokaryotes in high-temperature environments and could be used as one indicator of thermal adaptation for prokaryotes.

## Materials and methods

### Selection and extraction of DNA sequences

We downloaded the prokaryote growth temperature data (e.g. minimum temperature (T_min_), optimal temperature (T_opt_), and maximum temperature (T_max_)) from the TEMPURA database (http://togodb.org/db/tempura), which contains curated information for 8645 prokaryotes (549 archaea and 8096 bacteria). The complete genomic DNA sequences and their corresponding annotation files were obtained from the Genome Database of the National Center for Biotechnology Information (NCBI, https://www.ncbi.nlm.nih.gov/genome). To ensure the reliability and completeness of our dataset, we included only completely assembled genomes in our analysis. Using the links to the NCBI Taxonomy database and the taxonomy IDs provided by TEMPURA for each prokaryotic strain, we selected 681 bacterial genomes for analysis, excluding 155 archaea due to their limited number, which could result in a lack of significant correlations between genomic GC content and T_opt_ [28]. The GC contents, genome sizes, and genome accession numbers are provided in Supplementary Table S1. Information on 16S rRNA sequences was sourced from the TEMPURA database, and the sequence length and GC content were calculated (Supplementary Table S2). Prokaryotes were categorized into four groups based on their T_opt_: psychrophiles (T_opt_ < 20°C), mesophiles (20°C ≤ T_opt_ < 45°C), thermophiles (45°C ≤ T_opt_ < 80°C), and hyperthermophiles (T_opt_ ≥ 80°C).

### Identification of G4s in genomic features

QGRS Mapper (https://bioinformatics.ramapo.edu/QGRS/index.php), which uses a predefined motif pattern to identify potential G4 sequences and provides detailed annotations of G4 motifs in specific genes or regions [34], was used for the identification of G4 motifs in the 681 16S rRNA encoding sequences. The default parameters for QGRS Mapper were set as follows: QGRS max length: 30, min G-group size: 2, and loop size: from 0 to 36, with a specific loop search string. Genomic G4 distribution patterns were determined by G4Hunter algorithm (https://bioinformatics.ibp.cz), which is more suitable for genome-wide scans [35, 36]. The frequency of G4 motifs was calculated by dividing the number of G4 sequences by the total length. Specifically, the G4 frequency in the genome was calculated by dividing the total number of G4 sequences by the entire genomic length. Conversely, for the 16S rRNA regions, the G4 frequency was determined by dividing the number of G4 sequences by the total length of the 16S rRNA encoding sequences. G4 stability was assessed using both the G4Hunter score for genomic sequences and the QGRS score for 16S rRNA sequences.

### Phylogenetic tree construction

The exact Taxonomy ID (taxid) for each analyzed group was obtained from the NCBI Taxonomy Database using the Taxonomy Browser. Phylogenetic trees for the 681 bacterial genomes and 12 *Thermotoga/(Pseudo)thermotoga species* ((*Pseudo)thermotoga elfii*, (*Pseudo)thermotoga lettingae*, (*Pseudo)thermotoga profunda*, (*Pseudo)thermotoga hypogea*, (*Pseudo)thermotoga caldifontis*, (*Pseudo)thermotoga thermarum*, *Thermotoga naphthophila*, *Thermotoga petrophila*, *Thermotoga str. RQ2*, *Thermotoga str. RQ7*, *Thermotoga neapolitana*, and *Thermotoga maritima*) were constructed using the Neighbor-Joining method. These trees were generated with the ‘ape’ and ‘phangorn’ packages in R (https://www.r-project.org/) by analyzing 16S rRNA gene sequences. The distance matrix was calculated using the Kimura 2-parameter model to ensure accurate representation of evolutionary distances. To assess the reliability and statistical support of the phylogenetic tree branches, bootstrap analysis with one thousand replicates was conducted. The resulting phylogenetic trees, along with their bootstrap support values, were visualized using the Interactive Tree of Life platform (https://itol.embl.de/).

### Relationship between G4s and bacterial growth temperature

We employed phylogenetic generalized least squares (PGLS) regression to examine the relationships among GC contents (whole genome and 16S rRNA), growth temperatures (T_min_, T_opt_, and T_max_), and G4 attributes (frequency and score) using the “caper,” “ape,” and “nlme” packages following default parameters and Pagel's “lambda” correlation ∼1 [28, 37]. PGLS enables to detect phylogenetic relationships among species, thus controlling for shared evolutionary history that influences biological traits [38, 39]. Pearson correlation statistics were analyzed to explore the associations between GC content, growth temperatures, and G4 attributes. The *R* value was used to determine the strength of these correlations. The pairwise comparison analysis of G4 frequency and score across the four groups (psychrophiles, mesophiles, thermophiles, and hyperthermophiles) was performed using ANOVA following normality and Lognormality tests in GraphPad Prism (v5.02, GraphPad Software, Inc.).

### Sequence logo analysis

All 16S rRNA encoding regions for the 12 *Thermotoga/(Pseudo)thermotoga* species were uploaded into UGENE software for comprehensive analysis. The locations of G4 sequences were identified using ClustalW alignment within UGENE (https://ugene.net/). The alignment results were then visualized using Jalview software (https://www.jalview.org/). Each identified G4-forming sequence was analyzed using the RNAfold tool (v 2.6.3) to predict its secondary structure free energy, which was then used as a parameter to assess the stability of the G4s (http://rna.tbi.univie.ac.at//cgi-bin/RNAWebSuite/RNAfold.cgi). To further analyze the sequences, sequence logo analysis was generated from the aligned sequences using the WebLogo 3 tool (https://weblogo.threeplusone.com/).

### Circular dichroism analysis

Circular dichroism (CD) analysis was conducted using a J-1500 CD spectrometer (Jasco International, USA). All spectra were collected within a wavelength range of 220–350 nm, with a 1 nm step width, a 1 s response time, and a 200 mdeg/0.1 dOD scale. The CD spectra represent the average of three scans of the same sample taken at room temperature and are baseline-corrected for buffer signal contributions. DNA oligonucleotide sequences for G4 analysis and their respectively reversed sequences for i-motif analysis were synthesized by Integrated DNA Technologies, Inc. (Coralville, IA, USA) (Supplementary Table S3). The oligonucleotide sequences (10 μM) used for analyzing G4 structures were heated to 95°C for 10 min in 50 mM Tris buffer (pH 7.5) with or without 100 mM KCl, and then slowly cooled to room temperature over 4-h period. Similarly, DNA oligonucleotide sequences (10 μM) used for analyzing i-motif structures were heated to 95°C for 10 min in 50 mM Tris–acetate buffer at pH 4.1 and pH 8.0, followed by slow cooling to room temperature over 4 h period.

The CD melting test was conducted using a J-1500 CD spectrometer, set to a 1 nm step width, a 1 s response time, and a sensitivity of 200 mdeg/0.1 dOD (Jasco International, USA). The G4 samples (10 μM) were heated to 95°C for 10 min in 100 mM KCl and 10 mM lithium cacodylate buffer (pH 7.1). Buffers such as Tris, MES, HEPES, and MOPS have significant pKa shifts with temperature changes, which are inappropriate for *T_m_* analysis [40]. After heating, the G4 samples were gradually cooled to room temperature over a 4-h period. Similarly, C-rich (i.e. i-motif) samples (10 μM) were heated to 95°C for 10 min in 100 mM KCl and 10 mM lithium cacodylate buffer (pH 5.5), followed by slow cooling to room temperature over a 4 h period. TMPyP4 is a G4-stabilizing compound that binds to G4 structures, preventing their recognition by G4-binding proteins or other molecules [6]. To incubate with TMPyP4 (Frontier Scientific, USA), the compound was added to the annealed mixture at a final concentration of 20 μM and incubated overnight at 4°C, after which CD melting tests were conducted.

### Isothermal differential spectrum and thermal differential spectrum

Isothermal differential spectrum (IDS) and thermal differential spectrum (TDS) analyses for G4 and i-motif structures were carried out as previously described [41, 42]. Briefly, oligonucleotide solutions were prepared in 10 mM lithium cacodylate buffer under specified pH conditions. The solutions were heated at 95°C for 10 min and then allowed to cool slowly to room temperature over a 4-h period. Absorbance spectra were recorded using a Cary 60 UV/Vis spectrophotometer (Agilent Technologies, France) equipped with a Cary Peltier accessory, with a scan range of 320–220 nm, a scan rate of 600 nm/min, and automatic baseline correction. For G4 IDS analysis, after recording the initial spectra (unfolded, as no potassium was present), 100 mM KCl was added (folded), and the UV absorbance spectra were recorded after 15 min of equilibration, with dilution correction applied. For i-motif IDS analysis, spectra were recorded in two conditions: unfolded at pH 8.0 and folded at pH 5.5. Each IDS was calculated as the arithmetic difference between the unfolded and folded spectra. TDS data were obtained by recording absorbance spectra from 220 nm to 320 nm at 25°C for the folded G4/i-motif structures and after 10 min at 95°C for the unfolded structures. The TDS signature was determined by subtracting the folded structure spectra from the unfolded spectra, zero-corrected at 320 nm, and normalized to the maximum absorbance.

### Organism and growth conditions

*T. maritima* strain MSB8T and *T. elfii* strain SEBR 6459 were obtained from the Deutsche Sammlung von Mikroorganismen und Zellkulturen (DSMZ), Braunschweig, Germany. The *Thermotoga* medium (DSMZ medium 343) composition included 5.0 g soluble starch, 0.5 g KH_2_PO_4_, 2.0 mg NiCl_2_·6H_2_O, 20.0 g NaCl, 0.5 g yeast extract, 1.0 mg resazurin, 15.0 mL Wolfe's Mineral Solution, 250.0 mL artificial seawater, and 750.0 mL distilled water. All *Thermotoga* ingredients were dissolved except cysteine and sulfide, and the pH was adjusted to 6.5–6.8. The *Pseudothermotoga* medium (DSMZ medium 664) composition included 1.0 g NH_4_Cl, 0.3 g K_2_HPO_4_, 0.3 g KH_2_PO_4_, 0.2 g MgCl_2_·6H_2_O, 0.1 g CaCl_2_·2H_2_O, 0.1 g KCl, 10.0 g NaCl, 10.0 mL Modified Wolin's mineral solution, 0.5 g Na-acetate, 5.0 g yeast extract, 5.0 g trypticase peptone, 0.5 mL sodium resazurin (0.1% w/v), 1.5 g Na_2_CO_3_, 5.0 g Na_2_S_2_O_3_·5H_2_O, 4.0 g D-glucose, 0.5 g L-cysteine HCl·H_2_O, and 0.5 g Na_2_S·9H_2_O in 1000 mL distilled water. All *Pseudothermotoga* ingredients except carbonate, thiosulfate, glucose, cysteine, and sulfide were dissolved, and the pH was adjusted to 7.5. The two media were sparged with O_2_-free gas for 10 min to create an anoxic environment and then dispensed under the same atmosphere into 15 mL anoxic Hungate-type tubes (Chemglass Life Sciences, USA), filling them to 30–50% of their volume. The tubes were sealed with rubber stoppers and aluminum crimp seals and sterilized by autoclaving at 121°C for 30 min. After sterilization, the remaining reducing agents and chemicals were added from sterile, anoxic stock solutions prepared under O_2_-free gas.

Bacterial growth was monitored spectrophotometrically at 600 nm using a Beckman DU64 spectrophotometer (Beckman, USA). For inoculation, sterile 1 mL syringes with 20-G needles were used to introduce cells into the medium at an initial OD_600_ of 0.02 [43], unless specified otherwise. All cultures were incubated anaerobically at 80°C for *T. maritima* and 65°C for *T. elfii*. To investigate the potential impact of G4 structures on the 16S rRNA encoding gene, cultures were treated with 40 μM of the porphyrin compounds TMPyP4 and TMPyP2 (Frontier Scientific, USA). Growth levels were measured at 6- and 24-h post-treatment. For protein extraction, cells were disrupted by suspending them in 0.2% Triton X-100 in 50 mM Tris-HCl buffer (pH 7.5) containing 1 mM PMSF [44]. The lysate was clarified by centrifugation at 10 000 x*g*, and the protein content of the extracts was determined using the Lowry method [45].

### Quantitative real-time polymerase chain reaction validation

The bacterial culture was centrifuged at 6500 x*g* for 10 min at 4°C to remove the supernatant. A total of 500 μL of TRIzol reagent was added to the pellet, thoroughly mixed, and incubated at room temperature for 5 min. Then, 100 μL of anhydrous chloroform was added, and the tube was tightly sealed and shaken vigorously for 15 s to mix. The sample was allowed to stand at room temperature for 3 min, followed by centrifugation at 12 000 x*g* for 15 min at 4°C. The aqueous phase was carefully transferred to a new centrifuge tube, and 500 μL of isopropanol was added and gently mixed. After standing at room temperature for 10 min, the sample was centrifuged again at 12 000 x*g* for 10 min at 4°C. The resulting RNA pellet was washed with 1 mL of 75% ethanol and centrifugated at 7500 x*g* for 5 min. The RNA pellet was dissolved in an appropriate amount of RNase-free water (30–50 μL), and its concentration and purity were assessed using NanoDrop 2000 spectrophotometer (Thermo Fisher Scientific, USA).

For cDNA synthesis, the High-Capacity cDNA Reverse Transcription Kit (Thermo Fisher Scientific, USA) was used according to the manufacturer's protocol. Quantitative reverse transcription polymerase chain reaction (qRT-PCR) was performed using a reaction mixture containing cDNA, sequence-specific forward and reverse primers, and iTaq™ Universal SYBR® Green Supermix (Bio-Rad Laboratories, USA). The qRT-PCR program included an initial denaturation at 95°C for 3 min, followed by 45 cycles of denaturation at 95°C for 10 s and annealing/extension at 60°C for 60 s. A melting curve analysis was performed with 35–40 cycles of temperature increments from 65°C to 95°C (0.5°C increments for 2–5 s per step). The reference gene used was the heat-related chaperone gene *dnaK* [46]. Relative gene expression levels were calculated using the 2^^-△△^CT method, and primer sequences are provided in Supplementary Table S4.

## Results

### GC content and optimal growth temperatures in 681 bacterial species

**Figure 1.**
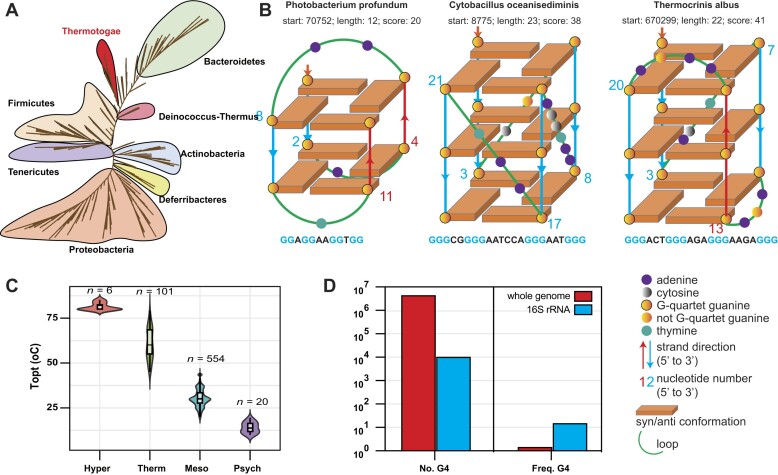
G4 motif distribution in 681 bacterial species. (**A**) Unrooted phylogenetic analysis of pathogen genomes based on 681 bacterial species, with different color ranges indicating 28 distinct bacterial phyla. (**B**) Schematic representation of G4 structures in representative bacterial species, illustrating anti-parallel (strands run in opposite directions), parallel (strands of the G4 run in the same direction), and hybrid (a mix of parallel and anti-parallel strands) arrangements. (**C**) Classification of bacteria into psychrophiles, mesophiles, thermophiles, and hyperthermophiles based on optimal growth temperatures. (**D**) Number and frequency of G4 motifs in the genome and 16S rRNA regions.

We collected a total of 681 bacterial growth temperature datasets using the TEMPURA database and downloaded the corresponding genomic and 16S rRNA gene sequences from NCBI (Supplementary Tables S1 & S2). These 681 bacteria belong to 28 bacterial phyla, including Proteobacteria, Firmicutes, Bacteroidetes, Actinobacteria, Thermotogae, Deferribacteres, Tenericutes, and Deinococcus-Thermus (Fig. 1A). The genome sizes in the dataset range from 800 407 bp to 13 033 779 bp, with genomic GC contents varying from 23.9% to 74.1%. The T_opt_ values range from 10°C to 85°C.

We used three canonical G4 examples to illustrate the orientation and arrangement of the G-tetrads, along with the loop configurations in the selected bacterial genomes. For instance, anti-parallel, parallel, and hybrid G4 structures are proposed to be present in *Photobacterium profundum* (T_opt_ 10°C), *Cytobacillus oceanisediminis* (T_opt_ 37°C), and *Thermocrinis albus* (T_opt_ 85°C), respectively (Fig. 1B). A high G4 score (e.g. 41 for *T. albus*) can be assigned to structures with three or more G-tetrads, compared to lower G4 scores (e.g. 20 for *P. profundum*) with only two G-tetrads.

### G4 motif distribution in genomes and 16S rRNA encoding regions across temperature adaptations

**Figure 2.**
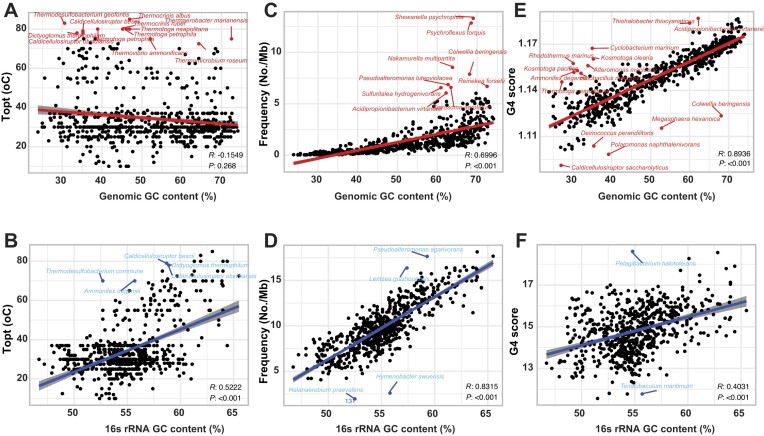
Relationship among optimal growth temperatures (T_opt_), G4 attributes, and GC content. Correlation analysis between bacterial T_opt_ and the GC content of whole genome (**A**) and 16S rRNA region (**B**). Correlation analysis between bacterial GC contents and the frequency and score of G4s in the whole genome (**C** and **E**) and 16S rRNA gene region (**D** and **F**). PGLS analysis was used to estimate the significance, and Pearson's *R* value was used to indicate positive or negative correlations.

**Figure 3.**
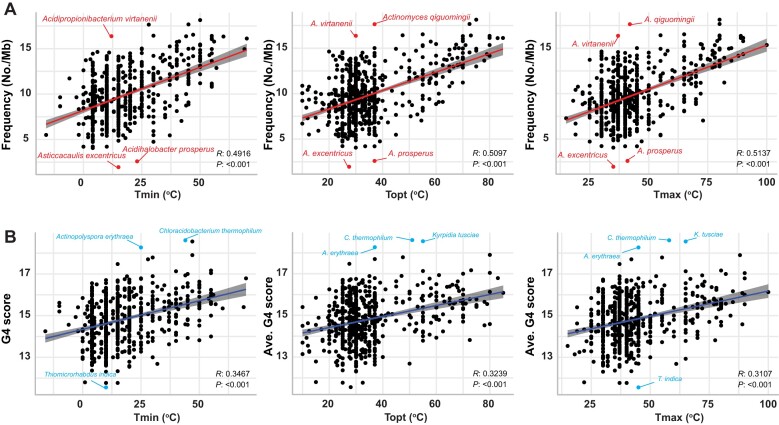
G4 Motif Distribution in 16S rRNA Genes. (**A**) Correlation analysis between bacterial growth temperatures (T_min_, T_opt_, and T_max_) and the frequency of G4s in the 16S rRNA region. (**B**) Correlation analysis between bacterial growth temperatures (T_min_, T_opt_, and T_max_) and the score of G4s in the 16S rRNA region. PGLS analysis was used to estimate the significance, and Pearson's *R* value was used to indicate positive or negative correlations.

We classified the 681 bacterial datasets into four groups based on their T_opt_: psychrophiles (T_opt_ < 20°C), mesophiles (20°C ≤ T_opt_ < 45°C), thermophiles (45°C ≤ T_opt_ < 80°C), and hyperthermophiles (T_opt_ ≥ 80°C). The majority of bacteria belong to mesophiles (554 species), while the psychrophiles, thermophiles, and hyperthermophiles groups contain 20, 101, and 6 species, respectively (Fig. 1C). We performed comprehensive scans of G4 motifs in both genomes and 16S rRNA encoding regions, identifying a total of 4160 695 G4 motifs in genomes and 9794 G4 motifs in 16S rRNA. Interestingly, the frequency of G4 motifs in 16S rRNA (9.79) was significantly higher than in genomes (1.37), indicating a higher potential for G4 formation in 16S rRNA (Fig. 1D). Through PGLS analysis, we identified a very weak positive correlation between genomic GC content and T_opt_, although linear correlation analysis suggested a very weak negative correlation (Fig. 2A). Interestingly, bacteria such as *Thermocrinis*, *Thermotoga*, and *Thermovibrio* have genomic GC contents not exceeding 50%, yet their T_opt_ values are above 70°C. It is unequivocally observed that the GC content of the 16S rRNA was significantly positively correlated with T_opt_ (Fig. 2B). In both genomes and 16S rRNA encoding regions, GC content was positively correlated with G4 frequency and scores, as high GC content facilitates the formation of G4 structures (Fig. 2C–F). PGLS analysis further revealed that only the G4 frequency and scores in 16S rRNA showed a strong positive correlation with T_min_, T_opt_, and T_max_ (Fig. 3A & B), whereas the G4 frequency and scores in genomes exhibited weak or no correlation with these temperature parameters (Supplementary Fig. S1).

### Elevated G4 frequency and stability in hyperthermophiles

**Figure 4.**
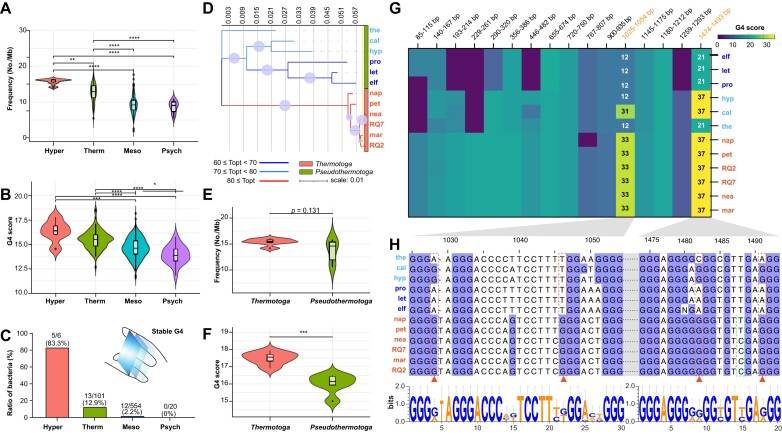
Evolutionary patterns and stability of G4 structures in hyperthermophiles. (**A**) Difference in G4 frequency across four groups: psychrophiles (psych), mesophiles (meso), thermophiles (therm), and hyperthermophiles (hyper). (**B**) Difference in G4 scores across the same four groups. (**C**) Ratio of stable G4 structures with at least three G-tetrads in psychrophiles, mesophiles, thermophiles, and hyperthermophiles. (**D**) Evolutionary patterns of 12 species classified into *Thermotoga* and *Pseudothermotoga*. (**E**) Difference in G4 frequency between *Thermotoga* and *Pseudothermotoga*. (**F**) Difference in G4 scores between *Thermotoga* and *Pseudothermotoga*. (**G**) Heatmap showing the presence pattern and scores of 16 G4 structures in *Thermotoga* and *Pseudothermotoga*. (**H**) Sequence analysis of two stable G4 structures in *Thermotoga*, with logo representation of consensus motifs found around positions 1025–1054 and 1474–1493 nts in *Thermotoga* and *Pseudothermotoga*. Abbreviations: nap: *T. naphthophila*, pet: *T. petrophila*, RQ2: *T. str. RQ2*, RQ7: *T. str. RQ7*, nea: *T. neapolitana*, mar: *T. maritima*, elf: *T. elfii*, let: *T. lettingae*, pro: *T. profunda*, hyp: *T. hypogea*, cal: *T. caldifontis*, the: *T. thermarum*, and mut: mutation.

ANOVA analysis indicated that in the 16S rRNA regions, the G4 frequency in hyperthermophiles was significantly higher than in thermophiles, mesophiles, and psychrophiles (Fig. 4A). Similarly, G4 scores were significantly elevated in hyperthermophiles and thermophiles compared to mesophiles and psychrophiles (Fig. 4B). High-scoring G4 structures are important indicators of G4 stability; we found that 83.3% (5/6) of hyperthermophiles possess three G4-tetrad structures (with scores exceeding 25), compared to 12.9% of thermophiles (13 species) and 2.2% of mesophiles (12 species). Psychrophiles, however, did not exhibit highly stable G4 structures (Fig. 4C and Supplementary Table S5).

### Evolutionary patterns and stability of G4 structures in the regions encoding 16S RNA

The variability in G4 frequency and scores in hyperthermophiles and thermophiles, prompted further analysis of their potential evolutionary patterns. *Thermotoga* serves as a valuable reference for thermal adaptation analysis due to its well-defined taxonomic classification and the broad range of microorganisms within the genus, exhibiting T_opt_ ranging from 60°C to 85°C. We selected 12 species from the genus *Thermotoga*, all with T_opt_ values above 60°C, based on data from the TEMPURA database and existing literature (Supplementary Table S6). Phylogenetic analysis supported the current classification of the *Thermotoga* genus, which primarily includes *Thermotoga* (i.e. *T. naphthophila, T. petrophila, T. str. RQ2, T. str. RQ7, T. neapolitana, and T. maritima*) and *Pseudothermotoga* (i.e. *T. elfii, T. lettingae, T. profunda, T. hypogea, T. caldifontis*, and *T. thermarum*) (Fig. 4D). *Thermotoga* species all have T_opt_ values exceeding 80°C, while *Pseudothermotoga* species have T_opt_ values below 80°C. There was no significant difference in G4 frequency between *Thermotoga* and *Pseudothermotoga* (*P* = 0.131) (Fig. 4E), but the G4 scores were significantly higher in *Thermotoga* (Fig. 4F and Supplementary Table S7). Consequently, we further analyzed the 16 conserved G4 structures expressed in the 12 species to explore specific G4 evolutionary trends and changes. All six *Thermotoga* species possessed these 16 G4s, except for a deletion of one guanine at position 783 nt in *T. naphthophila*, which prevented G4 formation (Fig. 4G and Supplementary Fig. S2). In contrast, all six *Pseudothermotoga* species had at least two GC rich regions that could not form G4 structures. We found that base mutations are a major factor influencing G4 scores in *Thermotoga* and *Pseudothermotoga*. In at least 11 of the 16 G4 structures, base mutations from adenine, cytosine, or thymine into guanine increased G4 scores in *Thermotoga* (Supplementary Fig. S2).

Notably, two G4 structures transitioned from unstable forms to stable forms with three G-tetrads, located at 1025–1054 nts and 1474–1493 nts. Comparing the logo sequences at these two positions in *Thermotoga* and *Pseudothermotoga* genomes revealed a conserved G-rich motif across all species (Fig. 4H). However, this ‘G-richness’ is more pronounced in *Thermotoga* species. For instance, guanines at positions 1028 and 1046 nts are dominant in *Thermotoga* genomes, while A or T are more common at these positions in *Pseudothermotoga* genomes. Similarly, positions 1482 and 1491 nts in *Thermotoga* genes also show near 100% prevalence of guanines. The other 14 G4s showed similar patterns with mutations that have a major or minor effect on G4 formation in *Thermotoga* (Supplementary Fig. S2). Overall, despite the consensus pattern of favoring G4 formation in both *Thermotoga* and *Pseudothermotoga* species, *Thermotoga* sequences are more conducive to G4 stability, as indicated by higher G4 scores and free energy assessments (Supplementary Fig. S3).

### *In vitro* spectroscopy reveals structural insights and stability of G4 and i-motif structures

**Figure 5.**
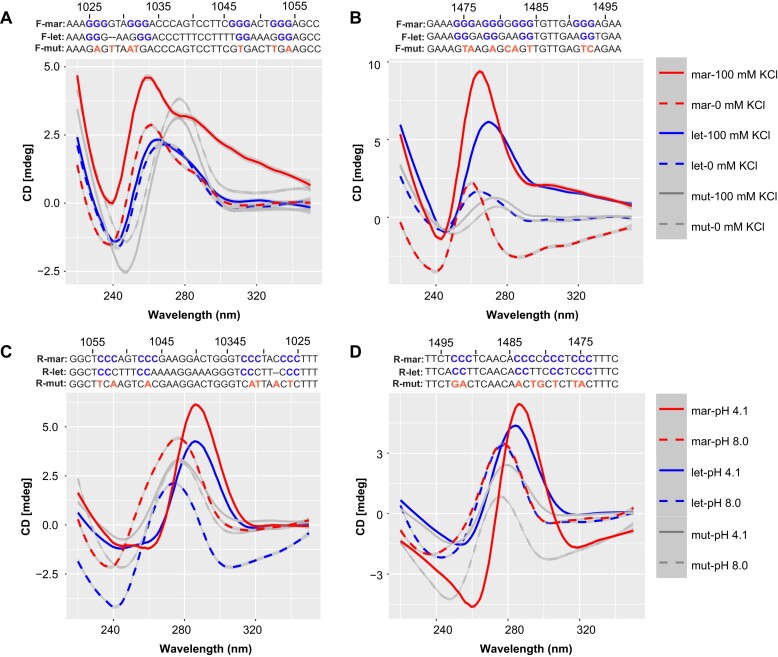
*In vitro* evidence for G4 and i-motif formation. (**A** and **B**) CD analysis of the forward ssDNA of the 1025–1054 and 1474–1493 nts regions in the absence or presence of 100 mM KCl, showing mutated nucleotides in the mutant sequence. (**C** and **D**) CD analysis of the reverse ssDNA of the 1025–1054 and 1474–1493 nts regions at pH 4.1 and 8.0, showing mutated nucleotides in the mutant sequence. Abbreviations: mar: *T. maritima*, let: *T. lettingae*, and mut: mutation.

We designed three pairs of DNA oligonucleotides for CD analysis: the first pair of sequences comes from *T. maritima* representing *Thermotoga* species, the second pair from *T. lettingae* representing *Pseudothermotoga*, and the third pair from mutated sequences (no G4 structure was detected using QGRS Mapper). For the F-strand of the *Thermotoga* group at 1025–1054 nts, a maximum peak was observed around 260 nm and a minimum peak at 240 nm (Fig. 5A). When 100 mM K^+^ was added, the *Thermotoga* group displayed the highest peak, indicating a typical G4 structure [47, 48]. In contrast, the *Pseudothermotoga* and mutated groups showed no change in peak values with or without 100 mM K^+^, suggesting that neither formed a G4 structure. The *Pseudothermotoga* group's CD absorption peak was the lowest among all groups due to a base deletion at position 1028. Mutating the guanine residues at positions −1026, −1028, −1031, −1032, −1047, −1052, and −1054 to A, T, A, T, T, T, and A, respectively, caused the maximum and minimum absorption peaks to shift to longer wavelengths, suppressing the K^+^-mediated enhancement of peak intensity compared to the *Thermotoga* group (Fig. 5A). Similarly, for the G4 structure at 1474–1493 nts, the addition of 100 mM K^+^ enhanced the absorption peaks in both the *Thermotoga* and *Pseudothermotoga* groups (Fig. 5B). However, in the mutant (with guanine residues at positions −1475, −1476, −1479, −1481, −1482, −1484, −1493, and −1494 mutated to T, A, A, C, A, T, T, and C, respectively) and under 0 mM K^+^ conditions, no enhancement of absorption peaks was observed (Fig. 5B).

For the R-strand of the *Thermotoga* group at 1025–1054 nts, the highest and lowest absorption peaks were around 290 nm and 260 nm, respectively (pH 4.1), with the *Thermotoga* group exhibiting a significantly higher peak than the *Pseudothermotoga* group (Fig. 5C). At pH 8.0, the maximum and minimum peaks shifted to 280 and 250 nm, respectively, consistent with previous analyses of i-motif structures by CD spectroscopy [48, 49]. Corresponding mutations also caused the maximum and minimum absorption peaks to shift to shorter wavelengths, blocking the H^+^-induced enhancement of peak intensity. Similarly, for the i-motif structure at 1474–1 493 nts, acidic conditions induced the formation of i-motif structures in both the *Thermotoga* and *Pseudothermotoga* groups, with the *Thermotoga* group showing significantly higher absorption peaks than the *Pseudothermotoga* group (Fig. 5D). Non-acidic conditions (pH 8.0) and mutant did not form i-motif structures.

We also analyzed the two G4 and i-motif structures in the hyperthermophile using IDS and TDS (Supplementary Figs. S4 and S5). Two G4 structures, particularly the one at nts 1474–1493 in *Thermotoga*, displayed distinct positive peaks around 240 and 275 nm, along with a notable negative peak near 295 nm. A similar pattern was observed for the reversed i-motif structure, which exhibited a strong positive peak at 240 nm and a negative peak at 295 nm. The G4 structure located at nts 1025–1054 appears to be less stable compared to the one at nts 1474–1493, likely due to the presence of long loops between its second and third G-tetrads. These IDS and TDS results for both G4 and i-motif structures are consistent with previous reports [41, 42]. However, in the *Pseudothermotoga* and mutant groups, no significant positive or negative peaks were detected for the G4 and i-motif structures at specific wavelengths. Additionally, CD melting temperature analysis was used to determine the *T_m_* values of G4 and i-motif structures *in vitro* (Supplementary Fig. S6). Both stable G4 structures in *Thermotoga* exhibited relatively high *T_m_* values, exceeding 60°C. The G4 stabilizer TMPyP4 increased the G4 melting temperature at both sites, while it either disrupted (nts 1025–1054) or decreased (nts 1474–1493) the i-motif melting temperature, consistent with previous analyses of CD melting tests [48, 50].

### Effects of TMPyP4 on growth and ribosomal gene expression

**Figure 6.**
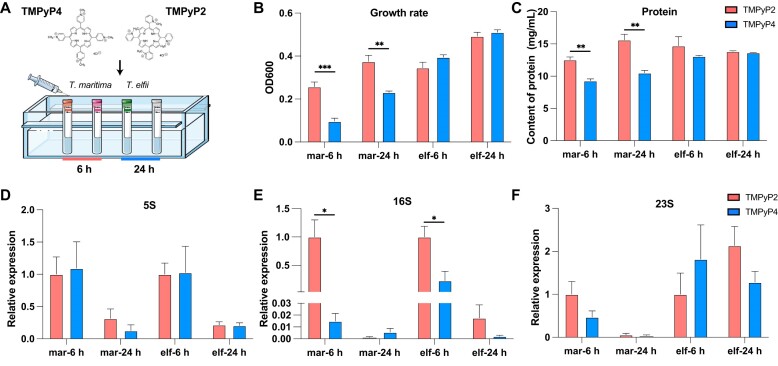
Effects of TMPyP4 and TMPyP2 treatments on *Thermotoga* and *Pseudothermotoga* growth, protein content, and ribosomal gene expression. (**A**) Schematic representation of the experimental setup, showing treatment with TMPyP4 or TMPyP2 and incubation for 6 and 24 h. (**B**) Bacterial growth measured as OD_600_ after 6 and 24 h. (**C**) Total protein content after 6 and 24 h of treatment. Relative expression levels of ribosomal genes 5S (**D**), 16S (**E**), and 23S (**F**) determined by qPCR at 6 and 24 h post-treatment. Data are shown as mean ± standard error (SE). Statistical significance is indicated as *P < 0.05, **P < 0.01, ***P < 0.001 (Student's t test). Abbreviations: mar: *T. maritima*, elf: *T. elfii*.

Bacterial growth was monitored at 6 h and 24 h post-treatment with 40 μM TMPyP4 or TMPyP2 to evaluate the impact of G4 stabilization on *T. maritima* and *T. elfii* (Fig. 6). TMPyP4 treatment significantly inhibited bacterial growth compared to TMPyP2 at both time points in *T, maritima* (*P* < 0.001 at 6 h and *P* < 0.01 at 24 h) but showed no alterations in *T. elfii* (Fig. 6B). Correspondingly, the total protein content was also reduced in the TMPyP4-treated cultures compared to TMPyP2 in *T. maritima* (*P* < 0.01 at both 6 and 24 h) (Fig. 6C). qPCR analysis of ribosomal genes revealed that TMPyP4 treatment caused a downregulation of the 16S rRNA gene at 6 h in the two bacteria (*P* < 0.05) (Fig. 6E), while the 5S and 23S rRNA genes (Fig. 6D and F) showed no significant differences at either time point. By 24 h, the expression levels of all ribosomal genes were not altered in the two treatment groups.

## Discussion

Our study provides a comprehensive analysis of the relationship between G4 structures and growth temperatures in prokaryotes by examining 681 bacterial genomes. This endeavor was inspired by the longstanding debate about the role of genomic GC content and its impact on thermal stability and adaptation. Our PGLS analysis suggested a very weak correlation between GC content and T_opt_, highlighting the complexity of thermal adaptation and suggesting that while GC content might play a role, it is not the sole factor. For example, species like *Thermocrinis albus*, *Thermotoga neapolitana*, and *Thermovibrio ammonificans* thrive at temperatures above 70°C despite having GC contents below 50%, indicating that other genomic features or regulatory mechanisms might significantly contribute to thermal stability. Therefore, despite the recent study claiming to resolve the previous contradictory observations and end the long debate by stating that prokaryotes growing at high temperatures have higher GC contents [25–28, 51, 52], but this correlation is very weak and likely applies only within certain temperature ranges, such as those of psychrophiles and mesophiles but not thermophiles and hyperthermophiles. However, the strong correlations observed in the G4 and 16S rRNA regions, which remain consistent regardless of the analytical approach used. Additionally, the relationship between GC content and growth temperature may be influenced by other significant evolutionary forces [53, 54]. For example, in the genus *Thermotoga* (T_opt_ range 60–85°C), the GC content ranges from 38.5% to 51.5%. Their relatively low GC content could be due to genome reduction and the loss of DNA repair genes, leading to decreased DNA repair efficiency [55, 56]. Despite this, the frequency of G4 structures in *Thermotoga* is high, likely playing a significant role in maintaining genome integrity and contributing to thermal adaptation.

Furthermore, our research indicates that while the G4 patterns in 16S rRNA encoding regions are strongly associated with T_opt_, the genomic G4 patterns do not show a significant correlation with T_opt_. This distinction is crucial as it points to a more specialized role of G4 structures in 16S rRNA in thermal adaptation, which is particularly relevant for hyperthermophiles and thermophile, where the need for stable ribosomal function is paramount due to the extreme conditions they inhabit [57, 58]. The 16S rRNA is essential for ribosome assembly and function, processes critical for protein synthesis and, consequently, for cell survival [59]. Formation of stable RNA G4 has been reported in large ribosomal RNA, where the authors suggested that these G4s are a common feature of the large subunit rRNA in human genome, potentially serving as switches between inter- and intramolecular G4s in rRNA tentacles [60]. Comparative studies have highlighted the role of G4 structures in the regulatory regions of genes in thermophilic archaea and bacteria, supporting the idea that these structures help regulate gene expression under thermal stress [31, 33]. Our findings add to this body of knowledge by specifically identifying the role of 16S rRNA G4 structures in thermal adaptation. This specificity is crucial because it points to a targeted adaptation mechanism where G4 structures in 16S rRNA potentially enhance the stability and functionality of ribosomes at higher temperatures. The presence of stable G4 structures in 16S rRNA likely provides a selective advantage to bacteria in extreme environments [61]. These structures might help maintain the integrity and efficiency of ribosome function under thermal stress, ensuring that protein synthesis can proceed uninterrupted [62, 63]. This is particularly important for hyperthermophiles, which thrive at very high temperatures and need highly stable molecular structures to survive. The elevated G4 frequency and stability in these organisms suggest a strong selective pressure favoring the formation of G4 structures in key genomic regions like those encoding the 16S rRNA genes.

Nature acts as a masterful and prolific chemist, creating numerous hostile niches that serve as ideal habitats for various thermophiles [17]. Among these, subsurface environments are particularly significant for the primary domain of the bacterial genus *Thermotoga* [64]. To date, more than 60 species of thermophilic bacteria and archaea have been identified, with certain species of the genus *Thermotoga* being of utmost importance [65]. Phylogenetic analysis supported the current classification of these genera, which was proposed to be split into two genera *Thermotoga* and *Pseudothermotoga* [66], and this classification was further corroborated by differences in G4 structure stability between the two groups. While there was no significant difference in the frequency of G4 structures between *Thermotoga* and *Pseudothermotoga*, the significantly higher G4 scores in *Thermotoga* suggest a stronger selective pressure for G4 stability in these high-temperature environments. Bacteria that thrive in high-temperature environments, such as those in the Deinococcus-Thermus group, are highly abundant in G4 structures [30]. This abundance is also related to their adaptation to other stress factors, such as radiation [67]. Environmental stressors like radiation can induce DNA damage, including double-strand breaks and oxidative lesions [68]. In response, organisms may evolve mechanisms to protect genomic integrity, including higher GC content, which is often linked to increased DNA stability under such conditions. Hence, some stable G4 structures could help prevent DNA damage by preventing DNA strand separation and serving as docking sites for DNA repair proteins [13]. In contrast, the *Pseudothermotoga* or even mesophile species, which thrive in relatively cooler environments, may experience limited selective pressure for G4 stability, leading to differences in the structural properties and functions of their G4 motifs. For example, in the mesophilic bacterium *E. coli*, which has an optimal growth temperature of around 37°C, G4 structures are less prevalent and often less stable compared to those in thermophiles [15, 69].

The CD melting temperature analysis revealed that the G4 structures in *Thermotoga* exhibit relatively high *T_m_* values. These results are consistent with previous findings, where *T_m_* values of 61.0°C and 67.3°C were reported for two potential G4 structures in other extreme thermophiles [30]. This close agreement further supports the notion that G4 structures in thermophilic organisms are highly stable and may play critical roles in maintaining genomic integrity under extreme conditions. It is speculated that specific DNA-binding proteins in the *Thermotoga* genus, such as those found in *T. maritima* and *T. neapolitana*, may contribute to enhancing the stability of DNA structures at elevated temperatures [70, 71]. Proteins with histone-like functions or Sac7d-like proteins, which are commonly found in thermophiles and extremophiles, have been shown to effectively stabilize DNA [71–73]. Such proteins could provide an additional layer of protection for G4 structures, ensuring their proper function even under thermal stress [72, 73]. Furthermore, the cytoplasm of thermophilic organisms typically contains high concentrations of potassium, sodium, and magnesium ions [74, 75]. These ions are known to stabilize G4 structures, suggesting that the *T_m_* values of G4s *in vivo* may be even higher than those measured *in vitro*. This increased stability in the cellular environment would further enhance the functional resilience of G4 structures, allowing them to withstand the high-temperature conditions typical of thermophilic habitats.

Our detailed analysis of 16 conserved G4 structures across these species revealed specific mutations that impact G4 formation. *Thermotoga* species consistently expressed these G4 structures, while *Pseudothermotoga* species showed multiple non-forming G4 structures due to specific mutations. Specifically, the G4 structures in *Thermotoga* species maintained intact G-tetrads at several key positions, such as guanine residues at positions 1474–1493 nts. In contrast, these positions in *Pseudothermotoga* species were often replaced by other bases, such as adenine or thymine, disrupting the formation of G4 structures. For example, mutations in *Pseudothermotoga* at critical guanine positions to other nucleotides led to the loss of G4 stability, preventing the formation of stable G4 structures. This evolutionary divergence indicates that *Thermotoga* species may have developed stable G4 structures as a response to high-temperature environments. Similar to the Levy jump model [76], there is an accidental jump in the evolution rate of GC content and growth temperature. The jump of GC content is significantly correlated with the change of growth temperature, and the formation of G4 structure may be induced during the skip process [28, 76]. Importantly, TMPyP4 treatment resulted in a significant downregulation of 16S rRNA gene expression in *T. maritima*, emphasizing the functional role of G4 structures in ribosomal gene regulation and thermotolerance. This suggests that the maintenance of G4 structures is essential for ribosomal activity, which may provide a selective advantage under extreme environmental conditions. On the other hand, *Pseudothermotoga* species, living in relatively cooler environments, may not experience the same selective pressure to maintain G4 stability. The conservation of G4-forming sequences and their functional relevance across species implies selective pressures that preserve these structural motifs throughout evolution. Similarly, the ancient sequencing of hepatitis B virus (HBV) revealed its long-standing association with humans, and analysis of G4 sequences in both ancient and modern HBV genomes showed a convergence in G4 frequencies with their hosts, suggesting an evolutionary ‘genetic camouflage’ strategy aiding in viral persistence and evasion of host defenses [77]. Furthermore, studies have revealed variations in G4 structures among species, including differences in sequence motifs, loop lengths, and structural stability, reflecting adaptations to specific genomic contexts and cellular environments [78, 79]. For example, over 5 million gains/losses or structural conversions of G4s can be caused by single-nucleotide variations in human genome, affecting transcription factor-binding sites and enhancers [78]. The evolutionary dynamics of G4s also extend to their interactions with regulatory proteins, nucleic acids, and small molecules, shaping complex regulatory networks and signaling pathways [14, 80].

Our study underscores the potential role of G4 structures in the thermal adaptation of bacteria, particularly through their presence and stability in 16S rRNA genes. The strong correlation between G4 stability and high growth temperatures suggests that these structures play a vital role in enabling bacteria to survive and thrive in high-temperature environments. This finding has significant implications for our understanding of bacterial adaptation and evolution. Future research should focus on experimentally validating the functional roles of G4 structures *in vivo*. This could involve exploring the impact of G4 structures on gene regulation, genome stability, and overall cellular function under different environmental conditions. Additionally, investigating the interplay between G4 structures and other genomic features, such as DNA repair mechanisms and regulatory proteins, could provide deeper insights into the molecular basis of thermal adaptation.

## Conclusion

In conclusion, our research underscores the pivotal role of G4 structures in the thermal adaptation of prokaryotes, with a particular emphasis on the 16S rRNA genes in thermophilic species. The strong positive correlation between G4 patterns in these genes and T_opt_ highlights their importance in maintaining ribosomal stability and function under extreme thermal conditions. The evolutionary analysis of *Thermotoga* and *Pseudothermotoga* species revealed significant differences in G4 stability, suggesting that stable G4 structures provide a distinct adaptive advantage in high-temperature environments. Other than 16S rRNA GC content, we also consider G4 patterns as one of the key indicators for the growth temperature of prokaryotes. Furthermore, the observed downregulation of 16S rRNA gene expression upon TMPyP4 treatment indicated the functional relevance of G4 stabilization in regulating ribosomal activity. Overall, our findings contribute to a deeper understanding of the molecular mechanisms underpinning thermal adaptation and offer promising directions for future research and innovation.

## Supplementary Material

gkaf042_Supplemental_Files

## Data Availability

The original temperature datasets analyzed in this study are available from the TEMPURA Database on prokaryotic growth temperatures (http://togodb.org/db/tempura). Supplementary materials accompanying this article provide further data and detailed information that support the study's findings and conclusions. Researchers and interested individuals can access these materials to explore and validate the study results.
