# Supplementary material for: G-quadruplex structures in 16S rRNA regions correlate with thermal adaptation in prokaryotes
Source: Nucleic Acids Res. 2025 Jan 30;53(3):gkaf042. doi: 10.1093/nar/gkaf042 (PMC11780868; doi:10.1093/nar/gkaf042)
Supplement: gkaf042_Supplemental_Files [file gkaf042_supplemental_files.zip › Figure s1-s6.pdf]

G-Quadruplex Structures in 16S rRNA Regions Correlate with Thermal Adaptation in Prokaryotes

Bo Lyu[a], Kangkang Niu[b], Deborah Anderson[c], Qili Feng[b], and Qisheng Song[a,*]

a Division of Plant Science and Technology, University of Missouri, Columbia, MO 65211, USA

b Guangzhou Key Laboratory of Insect Development Regulation and Application Research, Institute of Insect Science and Technology, School of Life Sciences, South China Normal University, Guangzhou 510631, China

c Department of Veterinary Pathobiology, University of Missouri, Columbia, MO, 65211, USA

*Corresponding author:

Qisheng Song; E-mail: songq@missouri.edu; Phone number: +1 573 882 979

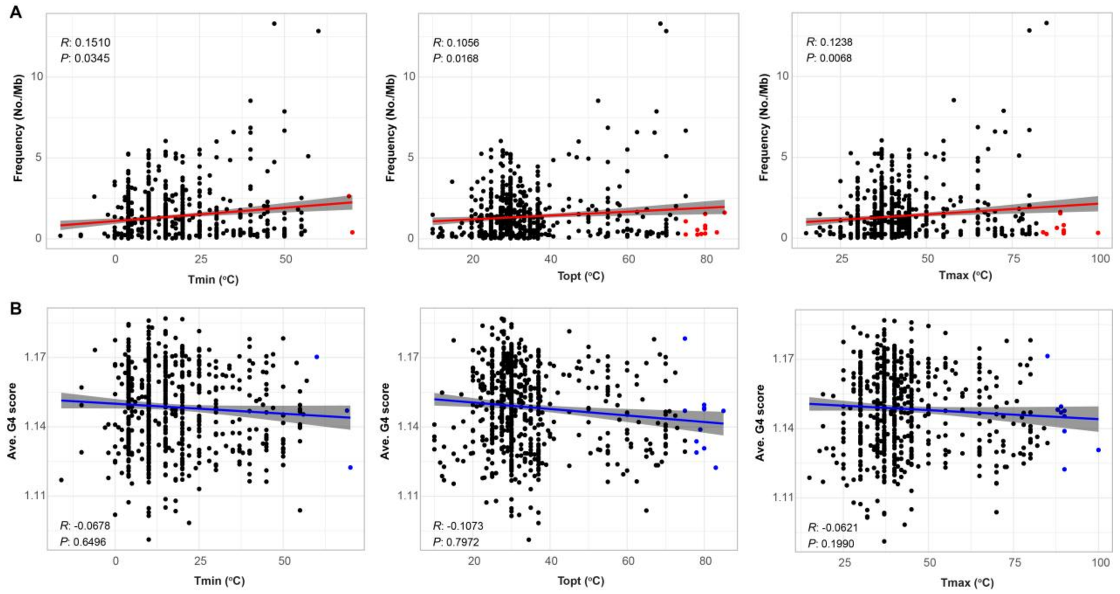

Figure s1. G4 Motif Patterns in Genomes and Their relationship with Growth Temperatures. (A) Correlation analysis between bacterial growth temperatures (T_{min}, T_{opt}, and T_{max}) and the frequency of G4s in the whole genome. (B) Correlation analysis between bacterial growth temperatures (T_{min}, T_{opt}, and T_{max}) and the score of G4s in the whole genome. PGLS analysis was used to estimate the significance, and Pearson's *R* value was used to indicate positive or negative correlations.

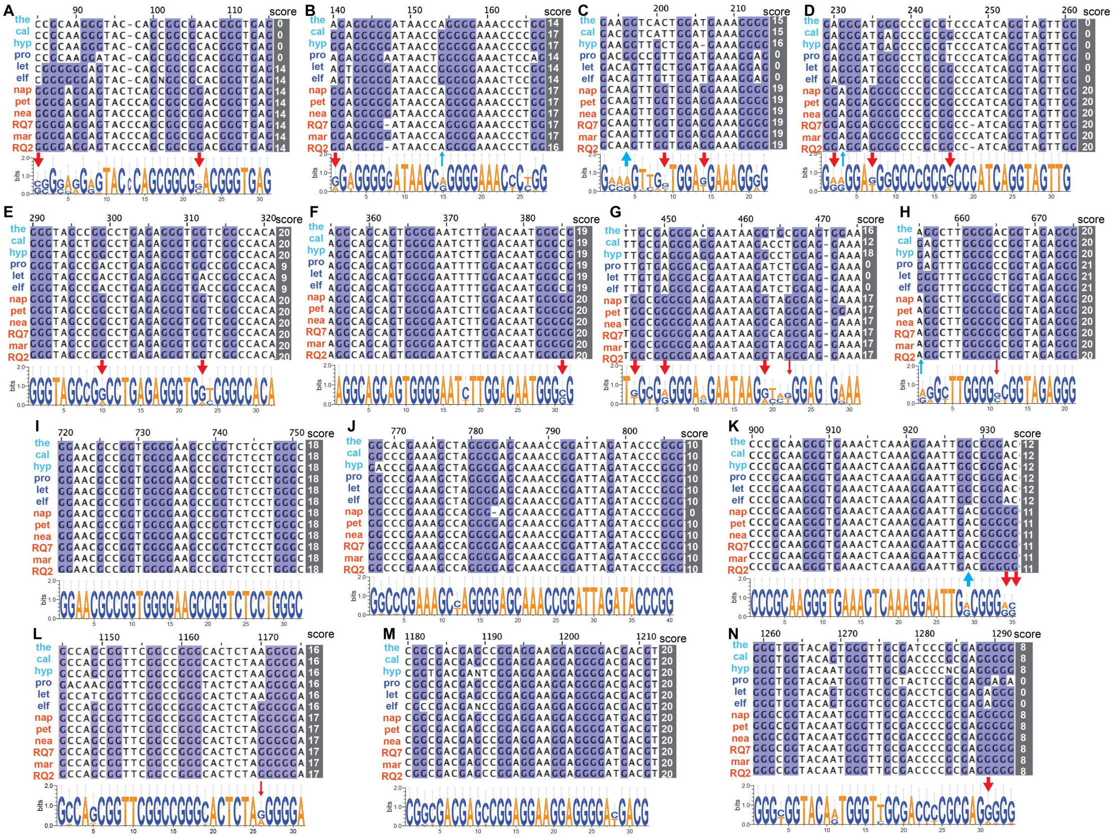

Figure s2. Sequence analysis and LOGO visualization of 14 G4 structures in *Thermotoga* and *Pseudothermotoga*. Sequence analysis and LOGO visualization of 14 G4 structures in Thermotoga and Pseudothermotoga. The 14 G4 consensus motifs are located at positions 85-115, 140-167, 193-214, 229-261, 290-320, 356-386, 446-482, 655-674, 720-750, 767-807, 900-935, 1145-1175, 1180-1212, and 1259-1293 nts. Red bold/regular dashes indicate guanine mutations (from adenine, cytosine, or thymine) that have a major/minor effect on G4 formation in *Thermotoga*. Blue bold/regular dashes indicate guanine mutations that have a major/minor effect on G4 formation in *Pseudothermotoga*. Abbreviations: nap: *T. naphthophila*, pet: *T. petrophila*, RQ2: *T. str. RQ2*, RQ7: *T. str. RQ7*, nea: *T. neapolitana*, mar: *T. maritima*, elf: *T. elfii*, let: *T. lettingae*, pro: *T. profunda*, hyp: *T. hypogea*, cal: *T. caldifontis*, the: *T. thermarum*, and mut: mutation.

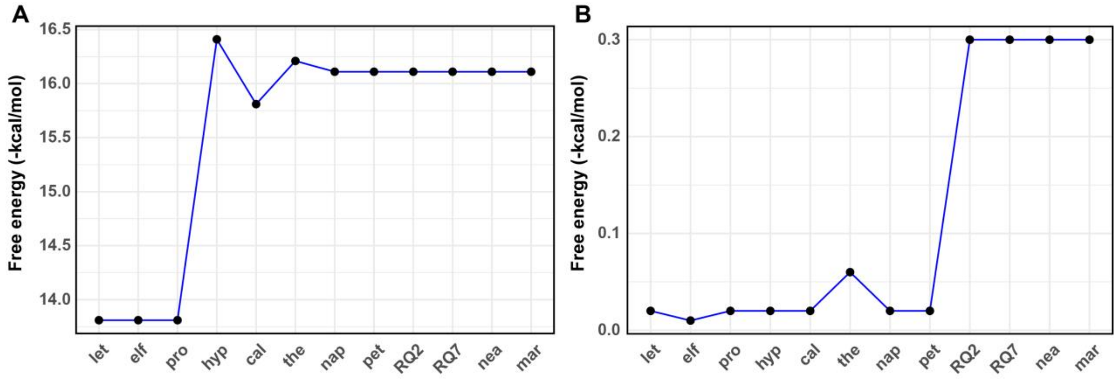

Figure s3. Prediction of Free Energy for Two Stable G4s Identified in *Thermotoga*. Prediction of free energy for two stable G4s identified in Thermotoga. (A) G4 structure at positions 1025-1054 nts. (B) G4 structure at positions 1474-1493 nts. Free energy was estimated using the RNAFold tool, with values influenced by nucleotide length and composition. For example, longer nucleotide sequences typically have more potential base-pairing interactions, which can contribute to lower (more negative) free energy values, indicating more stable structures. Abbreviations: nap: *T. naphthophila*, pet: *T. petrophila*, RQ2: *T. str. RQ2*, RQ7: *T. str. RQ7*, nea: *T. neapolitana*, mar: *T. maritima*, elf: *T. elfii*, let: *T. lettingae*, pro: *T. profunda*, hyp: *T. hypogea*, cal: *T. caldifontis*, the: *T. thermarum*.

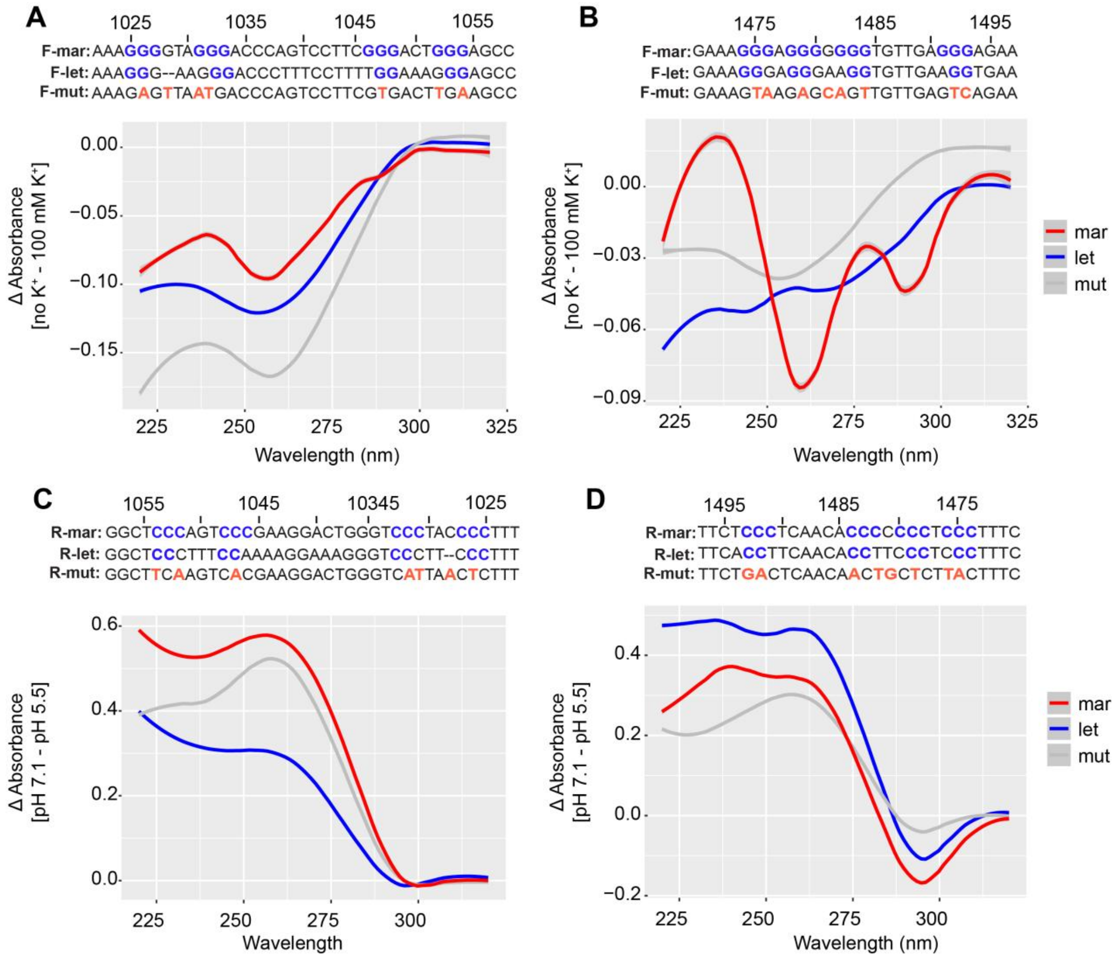

Figure s4. Isothermal difference spectra (IDS) of pre-folded G4 and i-motif structures. All spectra were recorded on a Cary 60 spectrophotometer (Agilent Technologies) in 1-cm pathlength quartz cuvettes (scan range: 320-220 nm; scan rate: 600 nm/min; automatic baseline correction). (A&B) IDS analysis of the forward ssDNA of the 1025-1054 nts and 1474-1493 nts regions in the absence or presence of 100 mM KCl, showing mutated nucleotides in the mutant sequence. (C-D) IDS analysis of the reverse ssDNA of the 1025-1054 nts and 1474-1493 nts regions at pH 4.1 and 8.0, showing mutated nucleotides in the mutant sequence. Abbreviations: mar: *T. maritima*, let: *T. lettingae*, and mut: mutation.

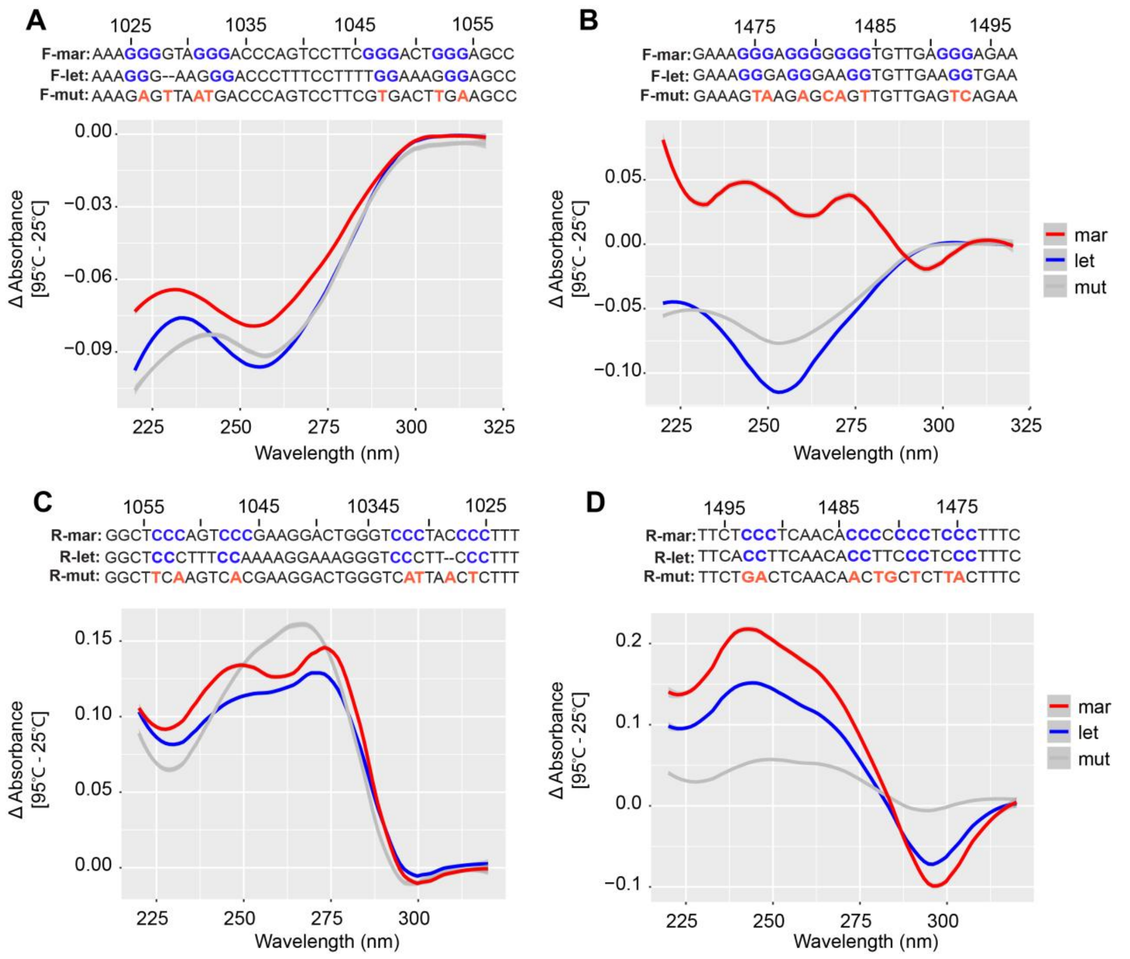

Figure s5. Thermal difference spectra (TDS) of pre-folded G4 and i-motif structures. All spectra were recorded on a Cary 60 spectrophotometer (Agilent Technologies) in 1-cm pathlength quartz cuvettes (scan range: 320-220 nm; scan rate: 600 nm/min; automatic baseline correction). (A&B) TDS analysis of the forward ssDNA of the 1025-1054 nts and 1474-1493 nts regions at high (95°C) and low (25°C) temperatures, showing mutated nucleotides in the mutant sequence. (C-D) TDS analysis of the reverse ssDNA of the 1025-1054 nts and 1474-1493 nts regions at high (95°C) and low (25°C) temperatures, showing mutated nucleotides in the mutant sequence. Abbreviations: mar: *T. maritima*, let: *T. lettingae*, and mut: mutation.

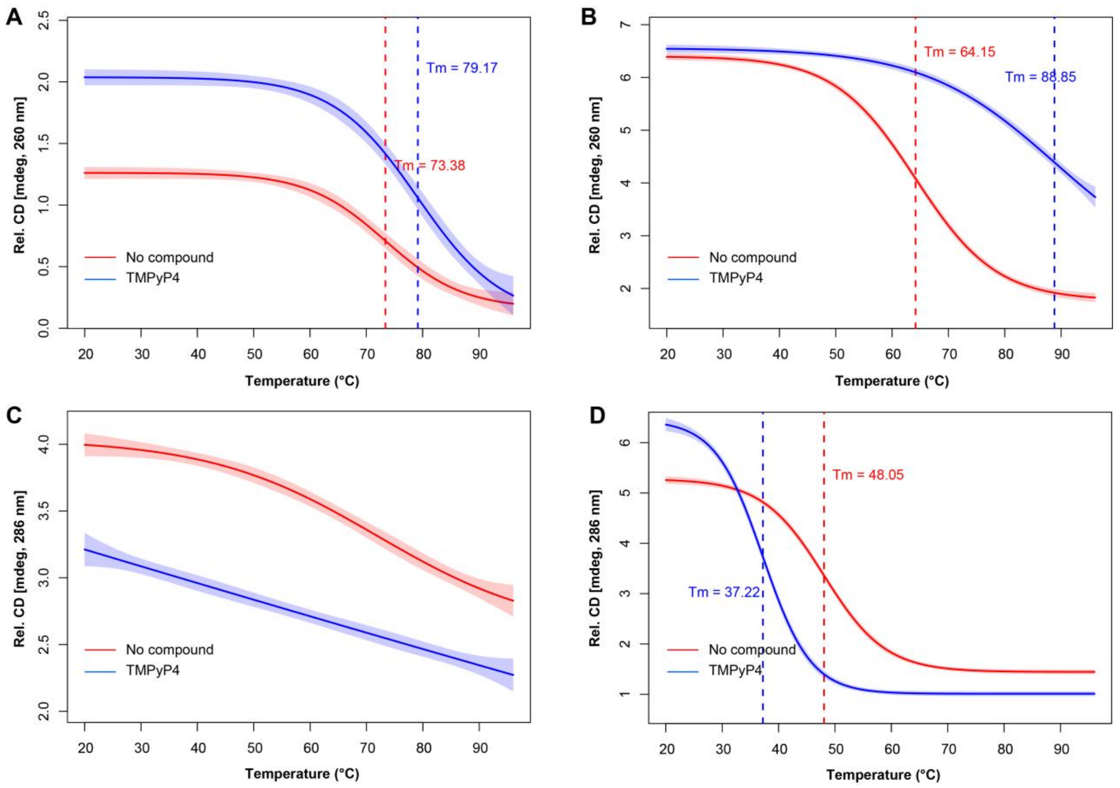

Figure S6. Circular dichroism analysis of the melting temperature of G4 and i-motif structures. Synthesized ssDNA oligonucleotides containing G4 (A&B) and i-motif (C&D) regions from the 1025-1054 nts and 1474-1493 nts sequences were heated at 95°C for 10 min in 10 mM lithium cacodylate buffer and then slowly cooled to room temperature over a 4 h period to allow the formation of G4 and i-motif structures. TMPyP4 was added to the solution to a final concentration of 20 μM and incubated overnight at 4°C, after which CD analysis was performed. The dotted lines indicate the melting temperatures.
